# Supplementary material for: Coinfection with Yellow Head Virus Genotype 8 (YHV-8) and Oriental Wenrivirus 1 (OWV1) in Wild Penaeus chinensis from the Yellow Sea
Source: Viruses. 2023 Jan 27;15(2):361. doi: 10.3390/v15020361 (PMC9964421; doi:10.3390/v15020361)
Supplement: Supplementary file 1 [file viruses-15-00361-s001.zip › Supplementary Figure S1.pdf]

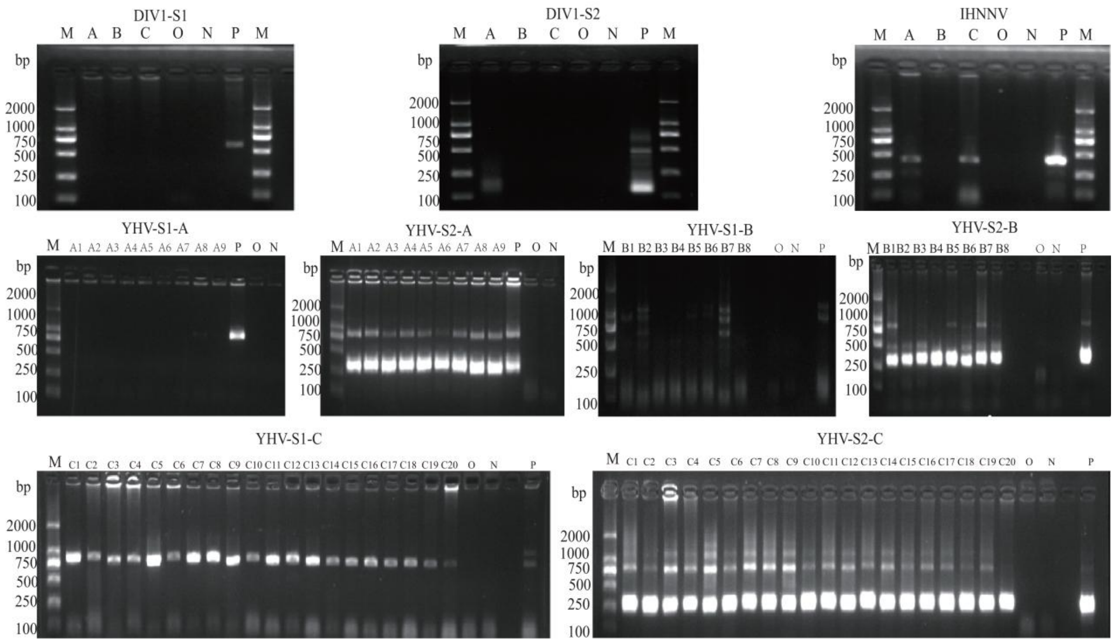

Figure S1. Agarose gel electrophoresis of positive pathogens by PCR detection. M: 2000bp Maker. A: Wild *Penaeus chinensis* caught in 2020. A1-A9: 20201124001-009. B: Wild *P. chinensis* caught in 2021. B1-B8: 20211126001-008. C: Wild *P. chinensis* caught in 2022. C1-C20: 20220309001-020. S1: The first step of nested PCR. S2: The second step of nested PCR. O: Blank control. N: Negative control. P: Positive control.
